# Impact of Hypertensive Disorders of Pregnancy on Maternal and Offspring Cardiovascular Health

**DOI:** 10.1007/s11886-026-02393-1

**Published:** 2026-07-24

**Authors:** Falastine Das, Susmita Parashar, Aparna Kulkarni

**Affiliations:** 1 Cohen Children’s Heart Center, Northwell Health, Cohen Children’s Medical Center, New Hyde Park, NY USA; 2 Division of Cardiology, Emory School of Medicine, Atlanta, GA USA; 3 Cohen Children’s Medical Center, 1111 Marcus Avenue, Suite M15, New Hyde Park, NY 11042 USA

**Keywords:** Hypertensive disorders of pregnancy, Maternal cardiovascular health, Offspring cardiovascular health, Intergenerational transmission of disease

## Abstract

**Abstract:**

Hypertensive disorders of pregnancy (HDP) affect 5–10% of pregnancies globally and are a leading cause of maternal and fetal morbidity and mortality. Beyond their acute obstetrics impact, HDP unmask a lifelong cardiovascular (CV) vulnerability in affected women and confer intergenerational risk to their offspring.

**Purpose of Review:**

In this review, we discuss the latest evidence regarding the CV risk profiles following HDP in the mothers and their offspring.

**Recent Findings:**

In mothers, HDP is associated with a 7.29-fold increased risk of new chronic hypertension (HTN) within 24 months postpartum and long-term risks of heart failure, coronary heart disease and stroke. Offspring exposed carry a 1.5-fold increased risk for adult HTN and increased risks for ischemic heart disease and stroke. We review evidence on potential pathophysiologic mechanisms including shared genetic, epigenetic programming and persistent immune dysregulation that contribute to the CV risk. We make recommendations on surveillance strategies in mothers and their offspring beginning in the immediate postpartum period and for lifelong follow-up. Management strategies of the CV risk factors, using latest guidelines are discussed. We suggest future directions for research and intervention.

**Summary:**

Integrated maternal-offspring CV care, women-specific risk prediction tools and equity focused research are needed to reduce the intergenerational burden of HDP.

## Introduction

Hypertensive disorders of pregnancy (HDP) are a group of disorders characterized by elevated blood pressure occurring before, during or after pregnancy. This includes chronic hypertension (HTN), gestational HTN, preeclampsia (PE)/eclampsia, and PE superimposed on chronic HTN [1]. These disorders are increasingly recognized not merely as transient complications of pregnancy but as sentinel events that unmask lifelong cardiovascular (CV) vulnerability in affected women and their offspring.

The HDP are a leading cause of maternal morbidity and mortality and can affect approximately 5–10% of all pregnancies globally [2]. Pre-eclampsia alone is responsible for more than 500,000 maternal deaths and over 500,000 fetal deaths worldwide [2]. The CV sequalae of HDP span from the immediate postpartum period through decades of subsequent life with an increased lifetime risk of cardiovascular disease (CVD) including chronic HTN, stroke, and cardiomyopathy, demanding a reframing of these conditions as chronic disease risk indicators rather than self-resolving obstetric events [3]. A retrospective study by Oliver-Williams et al. assessed the risk of CVD in nearly 2.5 million women affected by HDP and found that women with prior gestational HTN and PE had a greater cumulative incidence of total CVDs [4]. Not only did this association hold true decades after pregnancy, but the strength of this association increased with the number of affected pregnancies [4]. Similar findings were replicated by Kwak et al. who further delineated the risk of specific CVD outcomes versus the different subtypes of HDP [5]. The group found that not only were all subtypes of HDP associated with an increased risk of CV events, but that women with superimposed PE exhibited the highest composite risk compared to the other groups [5]. 

The long-term adverse effects of HDP are not limited to mothers only and can affect offspring outcomes in infancy, adolescence and ultimately adulthood. The maternal-placental-fetal interface refers to the complex, bidirectional interface between mother, placenta, and fetus during pregnancy, which is essential for fetal growth and development [6]. There is an increased risk of intrauterine growth restriction, preterm birth, perinatal death, neurodevelopmental delay, metabolic and CVD in the offspring of women affected by HDP [7]. Understanding these long-term consequences to both the mother and offspring is therefore essential in dictating the need for future screening and longer-term follow up.

In this review, we summarize the most recent evidence on maternal and fetal CV health in the context of HDP. We highlight the need for short and long-term CV surveillance in both mothers and offspring and propose a framework for how such follow-up could be implemented.

### Definitions and Key Mechanisms

Chronic HTN is defined as HTN occurring before the onset of pregnancy or before 20 weeks’ gestation, whereas gestational HTN (GHTN) develops after 20 weeks of gestation without proteinuria or organ dysfunction [1, 8]. Preeclampsia (PE) is a complex, multisystem disease characterized by HTN with involvement of at least one other organ system (renal, hepatic, hematologic or neurologic system). It is one of the most severe complications of pregnancy and can quickly progress to severe eclampsia or eclampsia (PE with seizures). Superimposed eclampsia occurs when women with pre-existing HTN develop features of PE after 20 weeks of gestation [1, 8]. Overall, superimposed PE carries a higher risk than either condition alone and confers a higher risk of development of long-term maternal CVD [1]. 

### Core Pathophysiological Mechanisms

The molecular mechanisms behind the development of HDP are complex and underscore the challenges in controlling and preventing the development of HDPs. At the core of the pathophysiology of HDP is abnormal placentation and placental ischemia where there is defective trophoblast invasion of the maternal decidua and impaired spiral artery remodeling [1]. This results in low circulating blood volume, high resistance uteroplacental circulation and ultimately placental hypoperfusion with chronic ischemia [9]. This in turn results in maternal endothelial dysfunction, oxidative stress, and an imbalance of pro-angiogenic and anti-angiogenic signaling [9]. These circulating factors lead to increased vascular sensitivity to vasoconstrictors with resultant HTN. Ultimately, the convergence of endothelial dysfunction, inflammation, and oxidative stress results in multi-organ involvement [1]. 

Immune and epigenetic dysregulation have also been linked to maternal disease progression and fetal programming. Differential methylation of cytosine-phosphate-guanosine islands and alterations in micro-RNA expressions that are associated with elevated CVD risk have been observed in women following PE [10]. Immune activation and inflammation play a pivotal role in the pathogenesis of HDP. Data from recent studies suggest that immune dysregulation can persist for years long after pregnancy, further offering insights into why HDP increase the risk of long-term adverse CV outcomes [11]. A study by Sabayev et al. utilized single cell mass cytometry and ex-vivo stimulation assays to characterize immune cell composition and function during pregnancy, in the immediate post-partum period, and extending several years thereafter. The study showed that compared to normotensive controls, women with HDP expressed features of immune dysregulation that persisted beyond pregnancy [11]. 

#### *Genetics, Epigenetics, and the Shared Maternal and Fetal Environmen*t

Early environmental exposures can induce epigenetic changes, defined as changes in gene expression without alteration in the underlying DNA sequence [9]. Emerging evidence highlights the role of epigenetics in abnormal placental development and immune dysregulation in the context of HDP. In addition, genetic predispositions contribute to both maternal and fetal susceptibility to future CVDs. This lifetime risk is further amplified by the shared maternal-fetal environment, which encompasses socioeconomic factors and lifestyle exposures such as obesity, smoking, and physical activity [1]. 

### Cardiovascular Outcomes in Mothers and Their Offspring

#### *Short-Term Maternal Cardiovascular Outcomes*

There is existing evidence to support the early risk of development of adverse CV outcomes in women affected by HDP. The highest CV risk occurs within the first year after delivery, with risk declining but remaining elevated over time [12]. In the immediate postpartum period, women with HELLP (hemolysis, elevated liver enzymes and low platelets) syndrome face increased risk of pulmonary edema, acute respiratory distress syndrome and renal failure [13]. In a longitudinal study by Ackerman-Banks et al. 119,422 pregnancies were examined to assess the cumulative risk of CVD within 24 months postpartum in those with and without HDP [14]. Within the first 24 months postpartum, women with HDP have a 2.81-fold increased risk of heart failure, a 2.9-fold increased risk of cardiomyopathy and a 1.43-fold increased risk of cerebrovascular disease compared to women without HDP [14]. Particularly striking is a 7.29-fold increased risk of new chronic HTN [14]. 

The temporal distribution of these events underscores the urgency of early surveillance: the highest proportion of first CVD occur during the first month after delivery, for cardiomyopathy (44%), heart failure (39%) and cerebrovascular disease (39%). The risk of chronic HTN after PE is 18 times higher in the first year, declining to 5 times higher at 5–10 years, while gestational HTN shows 12 times higher risk in the first year, declining to a 6 times higher risk at 5–10 years [12]. These data highlight that the first year postpartum is not a period of CV recovery but one of maximum CV risk.

#### *Long-Term Maternal Cardiovascular Outcome*s

The long-term CV burden following HDP is substantial and well-documented across multiple large epidemiological studies. Women with a history of PE face a 4-fold greater risk of heart failure, 2.5-fold greater risk of coronary heart disease, 1.8-fold greater risk of stroke and a 2.2-fold greater risk of CV death compared to women without HDP [15]. All hypertensive conditions in pregnancy are associated with approximately 5 times higher rates of chronic HTN and doubling of incident CVD [15]. 

The severity of HDP further stratifies long term CV risk. Meta-analysis show progressively stronger associations with CVD for moderate PE (odds ratio - OR 2.24) and severe PE (OR 2.74) [16]. Recent data from a large Korean cohort demonstrate that superimposed PE carried the highest risk of composite CV events (adjusted hazard ratio 2.93) [5]. Beyond CVD, women with PE history face 5 times greater risk of end-stage kidney disease (9 times for preterm PE), increased risk of diabetes, 3 times greater risk of vascular dementia and potentially elevated deficits in cognitive and motor function [15]. The risk of HTN becomes apparent within 10 years of the index pregnancy, even for women who had their pregnancy at age 20–30 years, making reproductive history an essential component of any CV risk assessment for women of middle age [15]. 

#### *Offspring Cardiovascular Outcomes*

The CV consequences of HDP extend beyond the mother to affect the next generation, their offspring. Offspring exposed in-utero to maternal HDP carry an elevated CV risk burden that manifests from childhood through young adulthood, constituting a significant intergenerational dimension of the HDP.

Meta-analyses consistently show that offspring exposed to PE in utero have systolic blood pressure (BP) 2–5 mm Hg higher and diastolic BP 1–4 mm Hg higher during childhood and young adulthood compared to those born to mothers without HDP [17, 18]. While, these differences may appear modest at the individual level, at the population level they translate to meaningfully elevated rates of chronic HTN. In utero exposure to HDP is associated with a 1.5-fold increased risk (HR 1.5, 95% confidence interval -CI 1.18–1.9) of adult HTN independent of maternal chronic HTN [19]. When both maternal HDP and chronic HTN are present, offspring carry a 2.4-fold increased risk, suggesting synergistic effects [19]. A large Norwegian cohort analysis reported that offspring exposed to PE have a 51% increased risk of HTN (adjusted HR 1.51, 95% CI 1.41–1.63) [20]. 

Offspring exposed to PE also have 0.36 kg/m^2^ higher BMI during childhood and adulthood than those without exposure [17]. A Norwegian study noted that an even higher BMI of 0.66 kg/m^2^ and 1.49 cm wider waist circumference in young adulthood in offspring of HDP than normotensive pregnancies [21]. Term PE is associated with higher non-HDL cholesterol (0.14 mmol/l) and triglycerides (0.13 mmol/l) than normotensive pregnancies [21]. 

A large Danish cohort study of 2.5 million individuals found that maternal HDP was associated with a 23% increased risk of early CVD in offspring (HR 1.23, 95% CI 1.19–1.26) from birth through age 40, with particularly elevated risk for HTN disease (HR 2.11) and myocardial infarction (HR 1.49) [22]. A Nordic multinational study of 8.5 million births demonstrated that offspring of mothers with PE have a 33% increased risk for of ischemic heart disease and 34% increased risk of stroke, independent of prematurity or small-for-gestation-age at birth [23]. Severe and early onset forms of PE confer the highest stroke risks (adjusted HR 2.55 for early onset vs. 1.18 for late onset PE) [23]. 

The interpretation of these associations is nuanced by emerging evidence on heritability. A 2023 Norwegian sibling study found that while population level analyses show increased risks of HTN, diabetes, and dyslipidemia in HDP exposed offspring, sibling comparisons show no significant associations, suggesting that shared genetic and familial factors may account for much of the observed risk rather than direct intrauterine programming [20]. A 2026 meta-analyses confirmed high heritability, with female offspring showing 72% increased odds of HDP (OR 1.72) and 90% increased odds of PE (OR 1.9) following maternal exposure to the same disorders [24]. This does not diminish the importance of monitoring the offspring but perhaps reframes the mechanisms underlying the risk. Nevertheless, offspring require surveillance regardless of the causal pathway.

### Implications for Clinical Practice

Timelines for evaluation, monitoring and follow-up of mothers with HDP and their offspring are detailed in Table 1.

**Table 1:** Recommendations for surveillance and management of women with hypertensive disorders of pregnancy and their offspring

Category	Timing	Recommended Action	Target / Goal	Guideline / Source
**MOTHERS — SHORT-TERM SURVEILLANCE & MANAGEMENT (0–12 months postpartum)**				
Blood Pressure Monitoring	1–2 weeks postpartum	Home BP monitoring; review antihypertensive medications; ensure BP < 130/80 mmHg before discharge	< 130/80 mmHg	ACOG 2019; ACC/AHA 2025
Postpartum Visit	4–12 weeks postpartum	Comprehensive cardiovascular risk assessment: history, physical exam, BMI, waist circumference, BP, heart rate	Identify persistent HTN, cardiomyopathy, metabolic risk	ACOG 2019; AHA 2021
Biochemical Screening	6–12 weeks postpartum	Fasting lipid profile, fasting glucose/HbA1c, urine protein-to-creatinine ratio, renal function	Detect dyslipidemia, diabetes, proteinuria	ACOG 2019
Antihypertensive Management	As needed postpartum	Continue or initiate antihypertensive therapy;	BP < 130/80 mmHg	ACC/AHA 2025
Lifestyle Counseling	At discharge and all postpartum visits	Counsel on DASH diet, sodium restriction, weight management, smoking cessation, alcohol limitation, physical activity	Return to pre-pregnancy weight by 12 months	AHA 2021; ACOG 2019
Exercise Intervention	6–12 weeks postpartum	Supervised or structured exercise program; consider digital/wearable activity programs	Improve endothelial function, metabolic indices	AHA 2021
Aspirin Prophylaxis	Subsequent pregnancies from 12 weeks’ gestation	Low-dose aspirin (81 mg/day) to reduce recurrent preeclampsia risk	Reduce recurrence of HDP	ACC/AHA 2025; ACOG
Breastfeeding Support	Immediate postpartum	Encourage breastfeeding; compatible antihypertensives (nifedipine, labetalol); associated with modest BP reduction	Support lactation; cardiovascular benefit	AHA 2021
**MOTHERS — LONG-TERM SURVEILLANCE & MANAGEMENT (> 12 months postpartum)**				
Annual Blood Pressure Check	Annually (lifelong)	Measure BP at least annually; use home BP monitoring where available; team-based care with telehealth	BP < 130/80 mmHg	ACC/AHA 2025
Cardiovascular Risk Assessment	Every 1–3 years	Apply sex-specific risk tools incorporating HDP history as risk enhancer; consider coronary artery calcium scoring in intermediate-risk women	Classify 10-year ASCVD risk; guide statin/BP therapy	ACC/AHA 2026 cholesterol; ACC/AHA 2025 HTN
Lipid & Metabolic Screening	2–3 years post-delivery, then every 3–5 years	Repeat fasting lipid panel, fasting glucose/HbA1c; screen for type 2 diabetes development	LDL per ASCVD risk category; HbA1c < 5.7%	ACOG 2019; AHA 2021
Statin Therapy	When indicated	Consider statin therapy in women with borderline or intermediate 10-year ASCVD risk, using HDP as a risk-enhancing factor	LDL reduction per ACC/AHA thresholds	ACC/AHA 2026 cholesterol guidelines
Renal Surveillance	Annually	Monitor blood pressure, urine protein-to-creatinine ratio, eGFR; increased vigilance in women with preterm preeclampsia (9× ESKD risk)	Detect CKD progression early	Chappell et al., Lancet 2021
Cognitive & Neurological Screening	As clinically indicated	Enquire about cognitive symptoms; consider neurological referral for women with history of eclampsia or severe preeclampsia	Early detection of vascular dementia, cognitive impairment	Chappell et al., Lancet 2021
Pregnancy History Documentation	At every primary care and cardiology visit	Record HDP subtype, gestational age of onset, severity, and recurrence as permanent CVD risk factor in medical record	Risk-informed lifelong care	AHA 2021; ACC/AHA 2025
Reproductive Counseling	Pre-conception visits	Counsel on recurrence risk; optimize BP, weight, and metabolic health before subsequent pregnancies	Reduce risk in future pregnancies	ACOG; ACC/AHA 2025
**OFFSPRING — SHORT-TERM SURVEILLANCE & MANAGEMENT (Birth to 5 years)**				
Neonatal Risk Documentation	At birth	Document maternal HDP history in neonatal and pediatric medical record; note subtype, severity, gestational age of onset	Flag for long-term cardiovascular surveillance	AHA 2021
Neonatal Cardiovascular Assessment	At birth and neonatal follow-up	Assess for complications of preterm birth and growth restriction; echocardiography if clinically indicated	Detect structural cardiac abnormalities	Clinical judgment
Blood Pressure Measurement	From age 3 years (annually)	Routine BP measurement at every well-child visit per AAP; interpret using age/sex/height normative tables	Identify early hypertension; BP within normal range for age	AAP 2017; AHA 2021
Growth & Metabolic Monitoring	Each well-child visit	Track BMI, weight, height; counsel on nutrition and physical activity; monitor for obesity	Healthy weight trajectory	Andraweera 2019
Lifestyle Counseling (Family-Based)	From infancy	Encourage heart-healthy diet, physical activity, limiting screen time; frame as family health — shared maternal-child risk	Primordial prevention of CVD risk factors	AHA 2021
**OFFSPRING — LONG-TERM SURVEILLANCE & MANAGEMENT (School age through adulthood)**				
Blood Pressure Monitoring	Annually through childhood and adolescence; at every clinical contact in adulthood	Continue annual BP measurement; repeat if elevated; initiate lifestyle interventions for high-normal or elevated BP	BP within age-sex-height percentile norms; <120/80 mmHg in adults	AAP 2017; AHA 2021
Lipid Screening	Age 9–11 years (universal); repeat age 17–21 years	Fasting lipid panel per AAP universal screening schedule; increased vigilance given elevated non-HDL cholesterol and triglyceride risk	LDL < 110 mg/dL in childhood; age-appropriate adult targets	AAP 2017; Andraweera 2019
Glucose & Metabolic Screening	Adolescence onward	Screen for insulin resistance, metabolic syndrome, and type 2 diabetes risk, particularly in overweight offspring	Fasting glucose < 100 mg/dL; HbA1c < 5.7%	AHA 2021
Stroke Risk Surveillance	Infancy and young adulthood	Heightened vigilance for cerebrovascular symptoms in infancy (highest stroke risk) and young adulthood (re-emergence of risk); prompt neurological evaluation if symptomatic	Early detection of cerebrovascular events	Yang et al., JAMA Network Open 2022
Ischemic Heart Disease Screening	After adolescence into adulthood	Assess cardiovascular risk factors at routine adult visits; document maternal HDP history as risk enhancer; consider formal ASCVD risk calculation from age 20	Early identification of high-risk adults for preventive therapy	Huang et al., PLoS Med 2021
Cardiovascular Risk Documentation	At transition to adult care	Transfer maternal HDP history to adult primary care and cardiology records; include subtype, severity, offspring birth history	Continuity of risk-informed care across pediatric-adult transition	AHA 2021; Saji et al. 2026
Lifestyle & Behavioral Counseling	Ongoing from childhood through adulthood	Reinforce heart-healthy diet, regular aerobic exercise, smoking avoidance, weight management; family-based approach in childhood	Optimal cardiovascular health (AHA Life’s Essential 8)	AHA 2021
**INTEGRATED MATERNAL–CHILD & SYSTEM-LEVEL RECOMMENDATIONS**				
Care Transition Planning	At postpartum discharge and 6-week visit	Establish warm handoff from obstetrics to primary care/cardiology for mothers; link to pediatrician with HDP documentation for offspring	Prevent loss to follow-up at obstetric-primary care transition	ACOG; AHA 2021
Multidisciplinary Maternal Health Clinic	Within 3 months postpartum	Refer to or establish multidisciplinary clinic (cardiology, maternal-fetal medicine, primary care, dietitian, social work) for women with HDP	Comprehensive lifetime ASCVD risk management	AHA Call to Action 2021
Telehealth & Remote Monitoring	Ongoing postpartum	Implement telehealth-enabled home BP monitoring; associated with improved BP control and cardiac structure at 6–9 months postpartum	Sustained BP control; improved cardiac function	ACC/AHA 2025
Community-Based Interventions	Postpartum and ongoing	Deploy community health workers, faith-based programs, peer support, and culturally tailored education targeting high-burden communities (especially Black women)	Reduce disparities in HDP surveillance uptake and cardiovascular outcomes	AHA 2021
Electronic Health Record Flagging	At point of care	Flag maternal HDP history in EHR for both mother and offspring records; trigger cardiovascular surveillance reminders at appropriate intervals	Systematic, guideline-concordant follow-up	System-level recommendation
Health Equity Monitoring	Ongoing	Track surveillance uptake and cardiovascular outcomes by race, ethnicity, and socioeconomic status; prioritize equitable access to postpartum and pediatric cardiovascular care	Reduce racial and socioeconomic disparities in HDP-related CVD	AHA 2021; ACC/AHA 2025

Abbreviations: ACOG, American College of Obstetricians and Gynecologists; ACC/AHA, American College of Cardiology/American Heart Association; AHA, American Heart Association; AAP, American Academy of Pediatrics; ASCVD, atherosclerotic cardiovascular disease; BMI, body mass index; BP, blood pressure; CKD, chronic kidney disease; DASH, Dietary Approaches to Stop Hypertension; eGFR, estimated glomerular filtration rate; ESKD, end-stage kidney disease; HbA1c, glycated hemoglobin; HDP, hypertensive disorders of pregnancy; HTN, hypertension; LDL, low-density lipoprotein

#### *Postpartum BP Monitoring, CVD Risk Assessment and Long-Term Follow up of Mothers*

The postpartum period represents a critical opportunity to initiate CV risk reduction strategies in women who have experienced HDP. The American College of Obstetrics and Gynecology (ACOG) recommends CV risk assessment within 3 months postpartum, that includes detailed medical history, physical examination including BP and BMI, and basic biochemical testing that includes lipid profile, diabetes screening, and urine protein-to-creatinine ratio [25]. The 2025 American College of Cardiology/ American Heart Association (ACC/AHA) HTN guidelines further recommend that mothers with resolved HDP have BP measured at least annually [8]. Home BP monitoring combined with a team-based care, medication self-management and telehealth is known to be associated with lower BP and improved cardiac structure and function at 6 and 9 months postpartum [8]. 

The 2025 ACC/AHA HTN guidelines, the AHA scientific statement on CV risk in pregnancy and adverse pregnancy outcomes and the 2026 ACC/AHA cholesterol guidelines all have formally recognized HDP as a sex-specific CV risk enhancer. They further endorse statin use and BP management for women with borderline or intermediate 10-year atherosclerotic CVD (ASCVD) risk [8, 26, 27]. The primary BP target is < 130/80 mm Hg. Continued surveillance within 2–3 years of delivery, with an initial CV risk factor screening are recommended [26]. Pharmacologic treatment should be considered if risk factors persist after 6–12 months of lifestyle modification [8]. All subsequent pregnancies should include prophylactic low-dose aspirin starting at 12 weeks’ gestation to reduce PE risk [26]. 

#### *Early BP Monitoring, CV Risk Screening and Lifestyle Counseling in Offspring*

The AHA recognizes that offspring of mothers with adverse pregnancy outcomes, including HDP, may benefit from CV risk screening and lifestyle modifications [26]. Early BP screening of children born after PE pregnancies may identify those requiring early interventions of preventive strategies to reduce later life CVD risk. The American Academy of Pediatrics (AAP) recommends one-time annual BP measurement at 3 years for all otherwise healthy children, and at every clinical encounter before age 3 years for children with specific risk factors such as small-for-gestation-age and prematurity < 32 weeks’ gestation [28]. Maternal HDP are recognized as perinatal factors that influence later BP in the AAP recommendations, however, they are not explicitly listed as an independent indication for earlier or more frequent BP screening. The stroke risk is highest in infancy and then re-emerges in young adulthood. Ischemic heart disease risk becomes more pronounced after adolescence, making the case for longitudinal surveillance [23]. 

### Interventions Targeting Maternal -Offspring Risk to Reduce Intergenerational CVD

Reducing maternal CV risk after HDP has the potential to attenuate intergenerational CVD burden, through direct intrauterine effects in future pregnancies or through shared household and lifestyle risk factors. Lifestyle modification is the cornerstone of postpartum CV risk reduction [26]. Women should be counseled to achieve pre-pregnancy weight by 12 months after delivery, as elevated BMI superimposed with a history of HDP is associated with significantly higher risk of developing chronic HTN [29]. A supervised 12-week exercise program in women with recent PE improved peripheral endothelial and autonomic function indices of metabolic syndrome [30]. A gamified digital health intervention with wearable activity trackers was found to be effective in increasing daily step counts over 12 weeks in postpartum mothers with recent HDP, in 55% of Black participants [31]. This has equity implications especially since higher HDP prevalence is known in Black women.

Dietary interventions should emphasize heart-healthy patterns including reduced sodium intake, increased fruits, vegetables and whole grains, limited alcohol and supplemental folate and iron [26, 29]. Community-based approached are essential to reach women who disengage from the healthcare system after delivery [30–32]. Multi-disciplinary maternal health clinics focusing on lifetime ASCVD risk, glucose control, weight management and dietary support have been implemented at several academic centers, representing a model for integrated postpartum care [32]. Long-term follow up should proactively track the risk of HTN, coronary artery disease, and stroke, incorporating pregnancy history as a permanent component of the CV medical record [26]. 

Perhaps the most transformative implication of the evidence reviewed here is the need for integrated maternal-offspring CV care systems. The siloed nature of obstetrics, pediatrics and adult medicine creates structural barriers to recognizing and managing HDP related intergenerational CV risk [26]. A family centered CV health approach would represent a paradigm shift in preventive cardiology. Such integration requires communication across specialties, shared electronic health records linking maternal pregnancy complications to pediatric and adult care, and public health frameworks that track CV risk across generations.

### Knowledge Gaps and Future Directions

Critical gaps remain, despite the substantial and growing body of evidence on HDP and CV outcomes that limit the translation of this knowledge into optimal clinical practice. The relative contributions of intrauterine programming versus shared genetic and familial factors to offspring CV risk remain incompletely resolved [20, 21]. The specific biomarkers involved in inflammation, oxidative stress, and angiogenesis that link HDP to futured CVD require further elucidation [10]. Mendelian randomization studies and large prospective cohorts with detailed genetic and epigenetic data are needed to disentangle the exact mechanisms and determine whether interventions targeting the intrauterine environment could meaningfully reduce offspring CV risk [10, 15]. 

While HDP are recognized as a CV risk enhancer, this recognition does not yet meaningfully improve discrimination in standard risk prediction models [33–35]. Development and validation of women-specific CV risk calculators that incorporate reproductive history, including HDP, gestational diabetes and other pregnancy related complications, is a research priority [2, 35]. Such tools would enable more precise risk stratification and facilitate earlier, more targeted preventive interventions [2, 34, 36]. 

Implementation of clear guideline recommendations for postpartum CV follow-up remains inconsistent. The transition from obstetric to primary care is a well-documented gap in the care continuum for women with HDP [2, 26]. Research is needed on scalable, effective care transition models, including telehealth, remote BP monitoring programs, particularly for underserved populations who bear disproportionate HDP burden [26, 32]. 

Randomized controlled trials of lifestyle and pharmacological interventions specifically in women with a history of HDP are largely absent [36]. Data on efficacy of dietary changes, exercise, and pharmacotherapy in preventing chronic HTN and CVD after PE remain inconsistent. Future clinical trials targeting this population are needed [34, 36]. 

The pediatric evidence base for offspring surveillance is severely underdeveloped [22]. No formal screening protocols with evidence-based timing, modalities and thresholds for intervention currently exist despite AHA recognition as a CV risk factor [26, 37]. Prospective studies tracking offspring from birth through adulthood, with rigorous CV phenotyping and intervention arms are needed to generate the evidence base for clinical guidelines [37]. 

Finally, health equity considerations are underrepresented in the current literature. Black women in the United States have substantially higher rates of HDP [38] and its sequalae yet are underrepresented in intervention trials and often face structural barriers to postpartum follow-up care [36]. Future research must prioritize diverse, representative cohorts and evaluate whether interventions are equally effective across diverse population groups [39]. Community based participatory research models and culturally tailored interventions hold promise for addressing these disparities and ensuring that the benefits of advancing knowledge in this field reach the populations most affected [38]. 

## Conclusions

Hypertensive disorders of pregnancy are far more than transient obstetric complications. They confer a lifetime CV risk trajectory in the mother and an inherited CV risk in the offspring. The evidence compellingly establishes that HDP CV consequences begin in the immediate postpartum period and extend through decades of subsequent life with persistently elevated risks of numerous CVDs and kidney disease as well as CV mortality. The formal recognition of HDP as a sex-specific CV risk enhancer in major guidelines is an important step, but implementation of systematic postpartum follow-up remains inconsistent, particularly in underserved populations.

The data also support meaningful risks of elevations in BP, HTN and early-onset CVD in offspring through relative contributions of direct intrauterine programming versus shared genetic and familial factors. Regardless of the causal mechanisms, the epidemiological signal is clear that offspring warrant longitudinal CV surveillance, beginning with early BP screening in childhood and continuing through adulthood.

Ultimately, HDP offer a uniquely actionable opportunity that predicts future CV risk in mother and offspring. Realizing this opportunity demands integrated maternal-child CV care frameworks, prospective studies with rigorous CV phenotyping across the life course, women-specific risk prediction tools incorporating reproductive history, and a health equity imperative that ensures the benefits of this evolving science reach the communities most affected. The reduction of HDP-related intergenerational CV burden is both a clinical and public health imperative.

## Key References

- Jones DW, Ferdinand KC, Taler SJ, Johnson HM, Shimbo D, Abdalla M, et al. 2025 AHA/ACC/AANP/AAPA/ABC/ACCP/ACPM/AGS/AMA/ASPC/NMA/PCNA/SGIM Guideline for the Prevention, Detection, Evaluation, and Management of High Blood Pressure in Adults: A Report of the American College of Cardiology/American Heart Association Joint Committee on Clinical Practice Guidelines. Journal of the American College of Cardiology. 2025;86(18):1567–678.
  - ○ This guideline published by a writing committee of many cardiovascular societies highlights the long-term risk associated with hypertensive disorders of pregnancy and provides key recommendations regarding diagnosis, assessment and management of these disorders in section 5.5.
- Moholdt T, Aye C, Bahls M, Crispi F, Ghossein-Doha C, Goossens E, et al. A call to action on pregnancy-related lifestyle interventions to reduce cardiovascular risk in the offspring: a scientific statement of the European Association of Preventive Cardiology of the European Society of Cardiology. European journal of preventive cardiology. 2025;32(10):798–810.
  - ○ This scientific statement reviews the mechanisms of adverse effects on the offspring from hypertensive disorders of pregnancy and other maternal co-morbidities. The statement makes recommendations for evaluation and follow up of offspring, in addition to maternal interventions directed toward offspring well-being.

## Data Availability

No datasets were generated or analysed during the current study.

## Abbreviations

HDP: hypertensive disorders of pregnancy
HTN: hypertension
CVD: cardiovascular disease
GHTN: gestational hypertension
PE: pre–eclampsia
OR: odds ratio
HR: hazard ratio
ASCVD: atherosclerotic cardiovascular disease
